# Lipidome of high-density lipoprotein is strongly perturbed in hyperalphalipoproteinemia resulting from a rare mutation in endothelial lipase

**DOI:** 10.1016/j.athplu.2025.07.001

**Published:** 2025-07-05

**Authors:** Livia Pisciotta, Marie Lhomme, Chiara Pavanello, Maharajah Ponnaiah, Arianna Strazzella, Alice Ossoli, Wilfried Le Goff, Laura Calabresi, Anatol Kontush

**Affiliations:** a Dietetics and Clinical Nutrition Unit, IRCCS-Polyclinic Hospital San Martino, Genoa, Italy; b Department of Internal Medicine, University of Genoa, Genoa, Italy; c IHU ICAN (ICAN OMICS and ICAN I/O), Foundation for Innovation in Cardiometabolism and Nutrition (ANR-10-IAHU-05), Paris, France; d Centro E. Grossi Paoletti, Dipartimento di Scienze Farmacologiche e Biomolecolari, Università degli Studi di Milano, Italy; e Sorbonne Université, INSERM, Institute of Cardiometabolism and Nutrition (ICAN), UMR_S1166, Paris, France

**Keywords:** HDL, Extremely high HDL-Cholesterol, Hyperalphalipoproteinemia, Endothelial lipase, Mutations, Lipidomics, Cardiovascular disease

## Abstract

Both low and extremely high concentrations of high-density lipoprotein (HDL)-cholesterol are associated with elevated cardiovascular risk. As extremely high HDL-cholesterol states of hyperalphalipoproteinemia (HALP) are rare, HDL particles in this condition remain poorly characterised. HALP may result from mutations in endothelial lipase (EL), a hydrolytic enzyme present in the circulation. Using targeted LC/MS-MS, we quantified the lipidome of HDL isolated from three female subjects with HALP caused by a heterozygous [c.526 G > T, p.(Gly176Trp)] variant of the *LIPG* gene and compared them with two healthy female controls. Our findings revealed a strongly perturbed HDL lipidome primarily involving enrichment in several phosphatidylcholine, phosphatidylserine, phosphatidylethanolamine plasmalogen, and lysophosphatidylethanolamine species. Some of these differences were equally observed in whole plasma. These alterations may reflect perturbations of lipoprotein metabolism secondary to defective lipid hydrolysis by EL and may have consequences for atheroprotective HDL functions.

## Highlights

• A rare heterozygous variant in the *LIPG* gene causes hyperalphalipoproteinemia.
• The lipidome of HDL is significantly altered in this condition.
• These lipidomic changes involve an enrichment of various phospholipids.
• Such alterations may impact biological activities of HDL particles.

## 1 Introduction

Low levels of plasma high-density lipoprotein-cholesterol (HDL-C) are a risk factor for cardiovascular disease (CVD) [1]. However, large-scale epidemiological studies reveal that subjects with extremely high levels of HDL-C paradoxically display elevated overall and cardiovascular mortality [2]. Hyperalphalipoproteinemia (HALP) is a metabolic condition characterized by highly elevated plasma levels of HDL-C above the 90th or 95th percentile of the general population. Primary HALP is a heterogeneous disorder, which can be transmitted as a dominant trait, due to rare loss-of-function (LOF) variants of genes encoding proteins involved in the metabolism of HDL. Although HALP can be associated with overt clinical manifestations and predisposition to CVD, the relationship between HDL-C levels and CVD risk in primary HALP is contradictory.

Mendelian randomization studies and genetic consortia data suggest that the association between HDL-C and CVD is non-causal. Therefore, it has been proposed that it is not the HDL-C itself that impacts atherosclerosis, but rather a biological function of HDL, or a process mediated by HDL, that cannot always be accurately measured through the assay of HDL-C [3,4]. This hypothesis implies that structure, composition and biological functions of HDL, including cholesterol efflux capacity, may be differentially affected by distinct mutations leading to HALP.

Endothelial lipase (EL) is a member of the triglyceride lipase family which includes lipoprotein lipase (LPL), hepatic lipase and pancreatic triglyceride lipase [5]. While LPL primarily hydrolyses triglycerides in triglyceride-rich, apolipoprotein (apo) B-containing lipoproteins, hepatic lipase acts on both triglycerides and phospholipids in all lipoprotein classes, and endothelial lipase breaks down preferentially HDL phospholipids. LOF variants of the *LIPG* gene encoding EL are associated with primary HALP. Indeed, individuals with heterozygous LOF variants display increased plasma HDL-C and enlarged HDL particles which apparently possess enhanced cholesterol efflux capacity from macrophages [6]. In addition, these variants are associated with reduced atherosclerosis. However, a more recent study of *LIPG* variants with various degrees of HALP seems to exclude a protective role of the high levels of HDL-C on CVD [7].

Recently, a HALP kindred was described in which three members were heterozygous for a novel variant of the *LIPG* gene [c.526 G > T, p.(Gly176Trp)] found to be deleterious in silico [8]. The probands had elevated plasma HDL-C, apolipoprotein (apo) A-I and phospholipids, as well as reduced pre-beta-HDL and EL concentrations. HDL from the carriers was enlarged, enriched in phospholipids and free cholesterol, while depleted of triglycerides, and possessed deficient cholesterol efflux capacity [8]. Precise molecular composition of HDL in the carriers of EL mutations however remains indeterminate.

In this study, we performed in-depth lipidomic analysis of HDL isolated from subjects with HALP caused by the [c.526 G > T, p.(Gly176Trp)] variant of the *LIPG* gene. Our findings reveal a strongly perturbed HDL lipidome which may have deleterious consequences for cardioprotective HDL functions.

## 2 Methods

Three female subjects with extremely high HDL-C levels who were heterozygous for endothelial lipase (EL) deficiency were recruited at the University of Genoa (Genoa, Italy). The subjects were carriers of the [c.526 G > T, p.(Gly176Trp)] variant of the *LIPG* gene described elsewhere [8]. The index case was a 24-year-old female who was referred to the Lipid Clinic of the University of Genoa for an extremely high plasma level of HDL-C. The 58-year-old proband's mother had a history of high levels of total cholesterol and HDL-C in the absence of CVD. The 27-year-old proband's sister also displayed extremely high HDL-C and suffered from polycystic ovarian syndrome. Two healthy, 50-years-old, normolipidemic, non-obese female subjects were recruited as controls. Informed consent was obtained from all study subjects. The study protocol was approved by the institutional human investigation committee of the participating institution.

Circulating levels of lipids and proteins were measured as described elsewhere [8]. Total HDL was isolated from 0.8 to 1.0 ml plasma samples by sequential ultracentrifugation using a table-top ultracentrifuge (Beckmann Optima MAX-XP, CA, USA) [9].

For lipidomic analysis, HDL (30 μg phospholipid) and plasma (10 μl) samples were supplemented with a mixture of deuterated internal standards (Avanti Polar Lipids, Alabaster, AL) and extracted as described elsewhere [9]. Lipids were measured using LC-MS/MS with a Prominence UFLC (Shimadzu, Tokyo, Japan) and QTrap 4000 mass spectrometer (AB Sciex, Framingham, MA, USA) [9]. Nine phospholipid and two sphingolipid classes were quantified, including phosphatidylcholine, lysophosphatidylcholine, phosphatidylethanolamine, phosphatidylethanolamine plasmalogen, lysophosphatidylethanolamine, phosphatidylinositol, phosphatidylglycerol, phosphatidylserine, phosphatidic acid, sphingomyelin and ceramide, which together comprised more than 200 molecular lipid species [9].

Between-groups differences in continuous and dichotomous variables were analysed using Student's t-test and Fisher's exact test, respectively. Significant lipid features were clustered using complete linkage with Pearson correlation distance matrix and plotted as a clustered heatmap. In these calculations performed using Multi Experiment Viewer (MeV) software version 4.9 (https://sourceforge.net/projects/mev-tm4/), a feature was considered significant at p < 0.05 after the Benjamini−Hochberg correction.

## 3 Results and discussion

Mean levels of HDL-C were greatly (+68 %, p < 0.05) elevated in the three EL-deficient female subjects (109 ± 17 mg/dl) relative to the two normolipidemic female controls (65 and 66 mg/dl). In parallel, mean plasma apoA-I was increased by +47 % (p < 0.05) in the carriers of the mutation (189 ± 15 mg/dl) vs. controls (126 and 132 mg/dl). Total cholesterol and LDL-C were equally elevated in the EL deficiency by +58 % and +61 % respectively (p < 0.05). By contrast, no difference in age, body mass index, non-HDL-C, triglycerides and apoB-100 was observed between the groups.

**Fig. 1:**
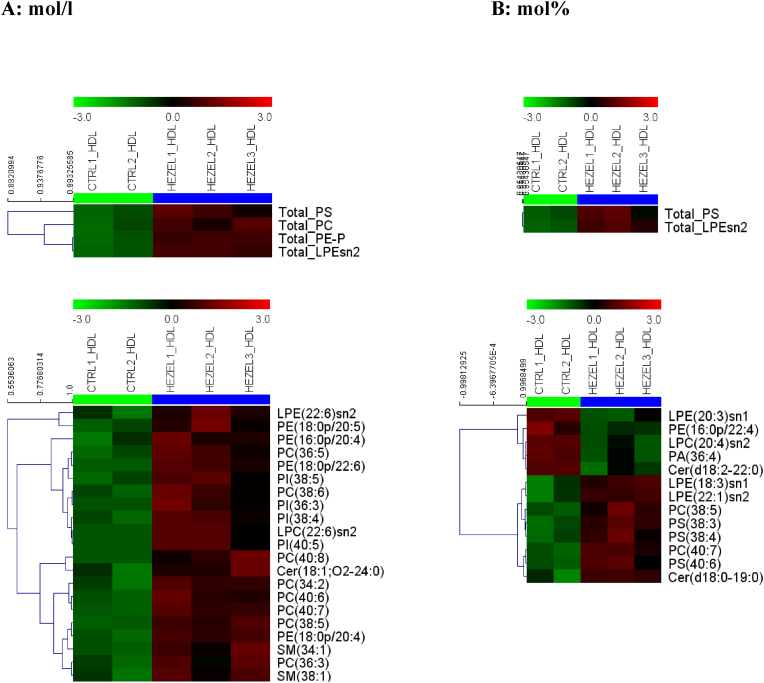
**Heatmap plots of the concentrations of total lipid classes and individual lipid species comprising the HDL lipidome in subjects with heterozygous EL mutations (HEZEL) relative to healthy normolipidemic controls (CTRL).** Lipid concentrations in HDL are expressed as mol/l plasma (A), or mol% of total HDL lipids calculated as a sum of all measured species (B). Cer, ceramide; LPC, lysophosphatidylcholine; LPE, lysophosphatidylethanolamine; PA, phosphatidic acid; PC, phosphatidylcholine; PE, phosphatidylethanolamine; PE-P, phosphatidylethanolamine plasmalogen; PI, phosphatidylinositol; PS, phosphatidylserine; SM, sphingomyelin.

Absolute concentrations of several lipid classes in HDL calculated per plasma volume were elevated up to 3.9-fold in the EL-deficient subjects vs. controls (Fig. 1, A). Indeed, plasma levels of total HDL phosphatidylcholine (+34 %), phosphatidylserine (+169 %), phosphatidylethanolamine plasmalogen (+56 %), and lysophosphatidylethanolamine (+289 %) were all increased in the carriers of the mutation. Lipid species belonging to different lipid classes were affected by the EL mutation to a variable extent. Indeed, while concentrations of eight HDL-associated phosphatidylcholine (+23 to +174 %), four phosphatidylinositol (+40 to +97 %) and four phosphatidylethanolamine plasmalogen (+30 to +219 %) species were elevated, concentrations of only two sphingomyelin (+51 to +69 %), one lysophosphatidylcholine (+103 %), one ceramide (+34 %), and one lysophosphatidylethanolamine (+497 %) species were increased (Fig. 1, A).

**Fig. 2:**
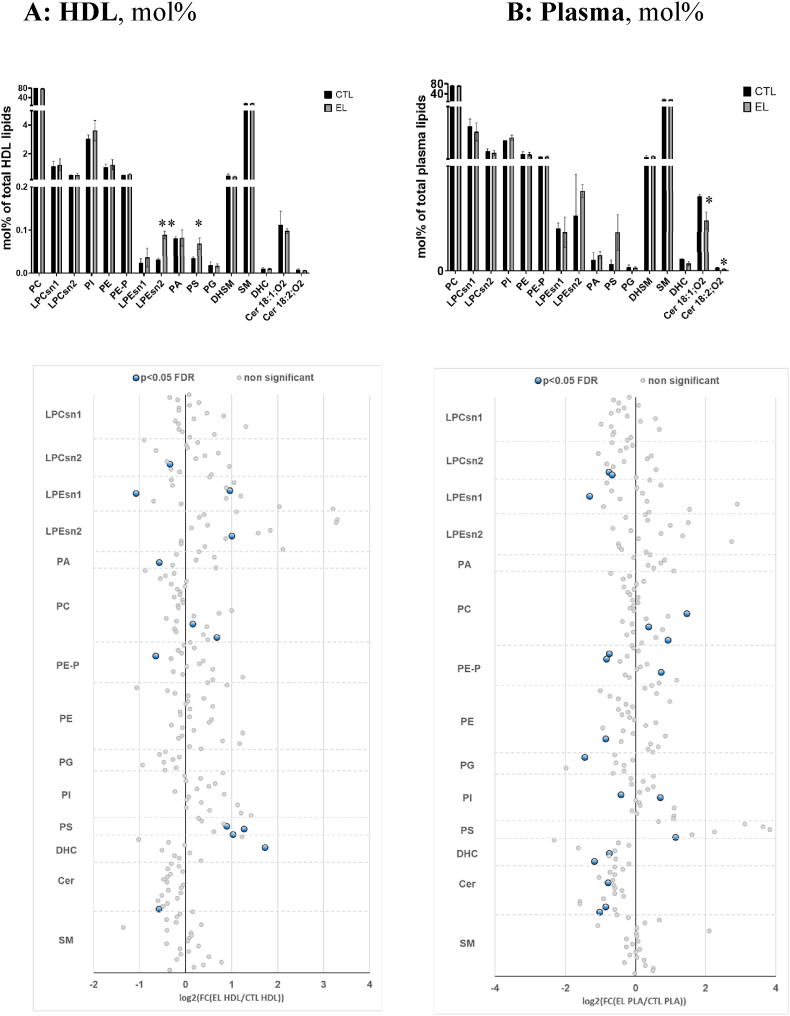
**Histogram and forest plots of the concentrations of total lipid classes and individual lipid species comprising the HDL (A) and plasma (B) lipidome in subjects with heterozygous EL mutations relative to healthy normolipidemic controls.** Lipid concentrations are expressed as mol% of total lipids calculated as a sum of all measured species. In the forest plots, the logarithm of the ratio of lipid concentrations measured in the EL mutated and control subjects is shown. Cer, ceramide; DHC, dihydroceramide; DHSM, dihydrosphingomyelin; FC, fold change; FDR, false discovery rate; LPC, lysophosphatidylcholine; LPE, lysophosphatidylethanolamine; PA, phosphatidic acid; PC, phosphatidylcholine; PE, phosphatidylethanolamine; PE-P, phosphatidylethanolamine plasmalogen; PG, phosphatidylglycerol; PI, phosphatidylinositol; PS, phosphatidylserine; SM, sphingomyelin.

When HDL lipid content was expressed as mol% of total lipids measured, differences between the groups became less pronounced. This result can be expected because, while absolute volumetric concentrations of major HDL components directly reflect elevated plasma levels of HDL, molar percentages reveal subtle relative changes in these components that do not necessarily parallel plasma concentrations. Among total lipid classes, HDL from the carriers was only enriched in phosphatidylserine (+96 %) and lysophosphatidylethanolamine (+183 %, Fig. 1, Fig. 2, A). In parallel, EL-deficient HDL revealed elevated abundances of two phosphatidylcholine (+11 to +60 %), three phosphatidylserine (from 85 to +140 %), two lysophosphatidylethanolamine (+94 to +100 %), and one ceramide (+230 %) species (Fig. 1, Fig. 2, A). Interestingly, HDL from the carriers was relatively depleted in several lipids, including one phosphatidylethanolamine plasmalogen (−36 %), one phosphatidic acid (−33 %), one lysophosphatidylcholine (−21 %), one lysophosphatidylethanolamine (−53 %), and one ceramide (−33 %) molecules (Fig. 1, Fig. 2, A).

**Fig. 3:**
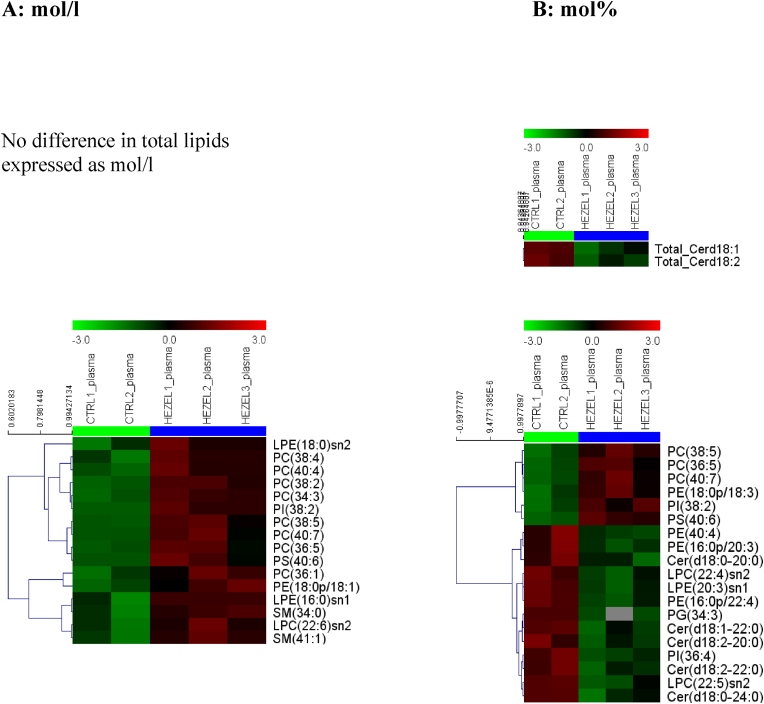
**Heatmap plots of the concentrations of total lipid classes and individual lipid species comprising the plasma lipidome in subjects with heterozygous EL mutations (HEZEL) relative to healthy normolipidemic controls (CTRL).** Plasma lipid concentrations are expressed as mol/l plasma (A), or mol% of total plasma lipids calculated as a sum of all measured species (B). Cer, ceramide; LPC, lysophosphatidylcholine; LPE, lysophosphatidylethanolamine; PC, phosphatidylcholine; PE, phosphatidylethanolamine; PG, phosphatidylglycerol; PI, phosphatidylinositol; PS, phosphatidylserine; SM, sphingomyelin.

Some differences in the lipid composition between EL-deficient and control subjects were equally observed in plasma (Fig. 3). Indeed, plasma levels of eight phosphatidylcholine (+23 to +174 %), two sphingomyelin (+51 to +69 %), two lysophosphatidylethanolamine (+ to +497 %), one lysophosphatidylcholine (+103 %), one phosphatidylinositol (+97 %), one phosphatidylethanolamine plasmalogen (+219 %) and one phosphatidylserine (+97 %) species were elevated in the carriers (Fig. 3, A). However, the concentration of no total lipid class was affected by the mutation.

When lipids were expressed as mol%, the pattern became more nuanced, with six lipid species, including three phosphatidylcholine (+30 to +176 %), one phosphatidylethanolamine plasmalogen (+66 %), one phosphatidylinositol (+63 %) and one phosphatidylserine (+119 %) enriched and 13 depleted in the carriers (Fig. 2, Fig. 3, B). Similar to observations in isolated HDL, the differences between absolute and relative concentration patterns likely reflected preferential association of the former with elevated plasma levels of HDL. Remarkably, five ceramide species were depleted (−39 to −56 %) in the carriers’ plasma as were two lysophosphatidylcholine (−37 to −41 %), two phosphatidylethanolamine plasmalogen (−40 to −43 %), one phosphatidylethanolamine (−45 %), one phosphatidylinositol (−25 %), one phosphatidylglycerol (−67 %), and one lysophosphatidylethanolamine (−60 %) species (Fig. 2, Fig. 3, B). In parallel, two ceramide subclasses revealed reduced abundance in the EL-deficient plasma (−33 to −45 %). As ceramides are predominantly carried in the circulation by large, non-HDL lipoproteins, this pattern might reflect decreased proportion of these particles in the carriers. Interestingly, two phosphatidylcholine 38:5 and 40:7 species were similarly affected both in HDL and plasma independent of the concentration basis used to express their concentrations, probably reflecting their predominant association with HDL [10]. These lipids bear a potential to serve as biomarkers of HALP resulting from EL mutations.

Molecular composition of HDL particles in HALP remains poorly characterized. Although several studies reported lipidomic composition of HDL in phenotypically defined, high HDL-C states, no data is available for genetically determined HALP, to the best of our knowledge.

HDL isolated from subjects with high HDL-C reveals elevated content of sphingomyelin, lysophosphatidylcholine and cholesteryl ester as compared to subjects with low HDL-C [11,12]. The enrichment of HDL in sphingomyelin and free cholesterol was equally observed, using NMR-based lipidomics, in subjects with high HDL-C relative to those with normal HDL-C [13]. In parallel, HDL content of triglycerides [11,13], ceramides [12], phosphatidylethanolamine plasmalogens and ether phosphatidylcholines containing polyunsaturated fatty acid residues [11] can be reduced in subjects with high HDL-C, while the content of ether phosphatidylcholines containing saturated fatty acids can be elevated [11]. When HALP is accompanied by CVD, this paradoxical phenomenon is associated with distinct differences in the HDL lipidome, which include depletion of phosphatidylcholine and phosphatidylinositol, as well as enrichment in sphingomyelin [14].

In the HALP kindred involving three heterozygous carriers of a novel variant of the *LIPG* gene [c.526 G > T, p.(Gly176Trp)], EL deficiency results in the alterations of the HDL composition, subclass distribution and function [8]. Our in-depth molecular lipidomic analysis reveal a strongly perturbed lipidome of HDL enriched in phosphatidylcholine, phosphatidylserine, phosphatidylethanolamine plasmalogen and lysophosphatidylethanolamine lipids, with altered composition of lysophosphatidylcholine, sphingomyelin, phosphatidylinositol and ceramide classes. These data most probably reflect disturbed lipolysis of HDL phospholipids by the mutant enzyme. Based on these findings, we hypothesize that lipidomic signatures may differ across distinct forms of HALP, reflecting their underlying genetic defects in *CETP, LIPC, APOC3, or SCARB1* - genes that impact fundamentally different pathways of HDL metabolism.

As all experimental studies, our research has certain limitations. Given the rarity of the *LIPG* mutation and the small sample size, this work should be considered a case report requiring validation in larger cohorts.

Our findings add specific molecular dimensions to the alterations of HDL in the genetic HALP, which may impact lipoprotein biology. For example, the accumulation of short-chain, proinflammatory lysolipids can reduce anti-inflammatory activity of HDL. In addition, the enrichment in sphingomyelin species observed herein can impair biological functions of HDL, acting via elevated surface rigidity [14]. This common feature, which is particularly expressed in HALP subjects with CAD, is consistent with the earlier studies of the HDL lipidome in subjects displaying high HDL-C [[11], [12], [13]]. Further studies are required to address the role of the lipidome for HDL function and CVD risk in HALP.

## Disclaimer

Given her role as Editor in Chief of Atherosclerosis Plus, Laura Calabresi had no involvement in the peer-review of this article and has no access to information regarding its peer-review.

## Funding

These studies were supported by National Institute for Health and Medical Research (INSERM; Paris, France), Sorbonne University (Paris, France), ICAN (Paris, France), Italian Ministry of University, Rome, Italy, Project # 2022JFCXH7, and Pfizer, USA, ASPIRE Program, Grant # 70215089.

## Declaration of competing interest

The authors declare the following financial interests/personal relationships which may be considered as potential competing interests: Anatol Kontush reports financial support was provided by National Institute for 10.13039/100018696Health and Medical Research (INSERM), Paris, France. Anatol Kontush reports financial support was provided by 10.13039/501100019125Sorbonne University, Paris, France. Marie Lhomme reports financial support was provided by 10.13039/501100020699ICAN, Paris, France. Laura Calabresi reports financial support was provided by Italian Ministry of University, Rome, Italy. Laura Calabresi reports financial support was provided by 10.13039/100004319Pfizer, USA. If there are other authors, they declare that they have no known competing financial interests or personal relationships that could have appeared to influence the work reported in this paper.
